# IoT-Enabled Adaptive Traffic Management: A Multiagent Framework for Urban Mobility Optimisation

**DOI:** 10.3390/s25134126

**Published:** 2025-07-02

**Authors:** Ibrahim Mutambik

**Affiliations:** Department of Information Science, College of Humanities and Social Sciences, King Saud University, Riyadh P.O. Box 11451, Saudi Arabia; imutambik@ksu.edu.sa

**Keywords:** IoT, sensor, traffic management, urban mobility optimisation, urban, smart cities, sustainability

## Abstract

This study evaluates the potential of IoT-enabled adaptive traffic management systems for mitigating urban congestion, enhancing mobility, and reducing environmental impacts in densely populated cities. Using London as a case study, the research develops a multiagent simulation framework to assess the effectiveness of advanced traffic management strategies—including adaptive signal control and dynamic rerouting—under varied traffic scenarios. Unlike conventional models that rely on static or reactive approaches, this framework integrates real-time data from IoT-enabled sensors with predictive analytics to enable proactive adjustments to traffic flows. Distinctively, the study couples this integration with a multiagent simulation environment that models the traffic actors—private vehicles, buses, cyclists, and emergency services—as autonomous, behaviourally dynamic agents responding to real-time conditions. This enables a more nuanced, realistic, and scalable evaluation of urban mobility strategies. The simulation results indicate substantial performance gains, including a 30% reduction in average travel times, a 50% decrease in congestion at major intersections, and a 28% decline in CO_2_ emissions. These findings underscore the transformative potential of sensor-driven adaptive systems for advancing sustainable urban mobility. The study addresses critical gaps in the existing literature by focusing on scalability, equity, and multimodal inclusivity, particularly through the prioritisation of high-occupancy and essential traffic. Furthermore, it highlights the pivotal role of IoT sensor networks in real-time traffic monitoring, control, and optimisation. By demonstrating a novel and practical application of sensor technologies to traffic systems, the proposed framework makes a significant and timely contribution to the field and offers actionable insights for smart city planning and transportation policy.

## 1. Introduction

Over the past few decades, urbanisation has accelerated dramatically, creating a range of complex challenges that demand innovative and sustainable solutions. Among the most urgent is urban traffic management, a key determinant of urban sustainability, economic performance, and public well-being. Congested roadways not only cause economic losses due to delays and inefficiencies, but also contribute significantly to environmental degradation, including higher greenhouse gas emissions and air pollution. The social consequences—such as increased stress and lowered quality of life—further reinforce the necessity of addressing congestion in modern cities [1,2,3].

A smart city framework has gained prominence as a viable means of tackling these issues, leveraging technologies like the Internet of Things (IoT), artificial intelligence (AI), and advanced data analytics [4,5,6]. Within this ecosystem, smart traffic management is widely recognised for its capacity to enhance mobility, ease congestion, and reduce transportation’s environmental footprint. Cities can now use various technologies to collect real-time data on traffic flow, vehicle counts, and journey times through embedded IoT sensors located at intersections and high-density corridors. This data is then used to optimise signal timing, reroute traffic, and enable responsive rather than static traffic control [7,8]. Such developments represent a paradigmatic shift from traditional, schedule-driven systems to adaptive and data-driven alternatives that dynamically respond to real-time conditions.

The evidence from recent studies has highlighted the economic and environmental stakes involved. For example, Hussain [9] estimated that congestion in major urban centres can lead to economic losses amounting to 2–5% of the GDP due to increased fuel consumption and productivity loss. In London alone, drivers lost an average of 227 h to traffic delays in 2022 [10]. From an environmental perspective, transport is a major contributor to CO_2_ emissions and local air pollutants, like nitrogen oxides (NOx) and particulate matter (PM), both of which are closely linked to urban traffic density. By reducing idle time and smoothing vehicle flow, well-designed traffic management systems can meaningfully cut these emissions [11,12].

One promising strategy for managing such complexity is the use of multiagent systems (MASs). These systems enable simulations of the interactions between independent entities within a transportation network—vehicles, pedestrians, and traffic signals—each programmed with specific behaviours and goals [13,14]. MAS approaches offer a way to examine emergent traffic patterns and assess how local actions scale up to system-wide outcomes. Prior studies have employed MASs to test routing strategies, model the effects of policy changes, and predict congestion hotspots [15,16,17]. However, the existing research often relies on either agent-based simulations without live data or IoT-enabled systems without sophisticated behavioural modelling.

What distinguishes this study from previous research is its combined focus on a simulation-based evaluation and the integration of real-time, IoT-derived traffic data into a multiagent system tailored to a complex real-world urban setting. While many existing models either simulate agent behaviour without live data or utilise IoT feeds without advanced agent-based modelling, this framework bridges the two by enabling a dynamic and behaviourally realistic response to actual traffic conditions. Although recent advances, such as V2V and I2V communication systems, enable highly granular, real-time data collection, most existing studies have not combined these capabilities with multiagent simulation frameworks that model autonomous, behaviourally diverse agents or incorporate predictive control mechanisms. In contrast, the inclusion of predictive control mechanisms further enhances the model’s practical relevance, allowing it to proactively manage congestion and emissions in ways that traditional reactive or static systems cannot.

This study aims to address the identified research gap by developing an MAS-based framework for assessing the performance of IoT-enabled smart traffic management systems. The model uses real-time data from sensors deployed at key urban points—intersections, arterial roads, and traffic-prone zones—and simulates the traffic dynamics under varying conditions, such as peak hours, roadwork, and special events. London was selected for the case study due to its complex transport network and chronic congestion, offering a rigorous testbed for the proposed system. Through region-specific inputs and customised performance indicators, the framework aims to deliver evidence-based insights for policymakers and planners.

A central innovation of this research lies in its incorporation of predictive traffic control algorithms within a multiagent simulation. These mechanisms use historical and live data to anticipate congestion before it occurs, enabling pre-emptive signal adjustments and adaptive route recommendations. For instance, if an intersection is forecasted to become a bottleneck, the system can revise signal phasing or divert traffic upstream. Similarly, drivers can receive updated directions in real time, based on the current and predicted conditions [18,19]. This predictive capability significantly enhances both user experience and system-level efficiency, reducing the average travel time and promoting sustainable commuting patterns.

Yet, despite the potential of IoT-enabled systems, their deployment raises a number of technical and ethical concerns. Chief among these is data governance: IoT systems routinely collect sensitive information, such as vehicle location and journey history, which must be protected from misuse or cyber threats [20,21,22]. Moreover, system reliability is contingent on the quality of the incoming data. Malfunctioning sensors or network disruptions can introduce errors, undermining traffic control decisions [23,24]. Accordingly, this study also examines the infrastructural and regulatory conditions necessary for its effective deployment, highlighting the best practices in system robustness and data stewardship.

Beyond the technical realm, its implementation success hinges critically on stakeholder engagement. Effective smart traffic systems require collaboration among local authorities, transport agencies, private-sector technology firms, and the general public. Citizen buy-in is particularly important; systems that are poorly understood or perceived as intrusive may provoke resistance. The study therefore stresses the importance of public education and transparent communication regarding system benefits, data use, and the anticipated impacts on daily travel behaviours [23,25]. An inclusive approach to system design and implementation is essential to ensuring its long-term viability.

By addressing these technical, social, and policy dimensions, the study makes a multifaceted contribution to smart city development. It showcases how simulation-based tools, informed by real-world data, can guide infrastructure design and transport planning in congested metropolitan settings. The findings are relevant not only to London but also to a broader class of cities facing similar mobility and sustainability challenges. The work underscores the added value of integrating IoT and MAS technologies to produce adaptive, context-sensitive traffic systems.

In summary, this research demonstrates how advanced simulations and real-time data can be jointly leveraged to improve urban mobility and environmental outcomes. The study’s hybrid framework represents a meaningful advance over prior approaches and provides a model for future innovations in intelligent transport systems. Its implications extend to urban planning, environmental management, and digital infrastructure strategy, offering a pathway toward smarter and more resilient cities.

## 2. Literature Review

This literature review synthesises the existing research on smart traffic management systems, focusing on both the technological innovations driving these systems and the socio-technical challenges surrounding their implementation. The review aims to contextualise the study’s MAS-based approach by examining recent developments in IoT, vehicle connectivity (V2V, I2V, and CAV technologies), and data-driven control methods, while also highlighting the implementation considerations, such as interoperability, governance, cost, and public trust. Taken together, these elements provide a multi-dimensional context against which the study’s proposed framework can be understood and evaluated.

### 2.1. IoT and Real-Time Traffic Frameworks

The evolution of smart traffic management (STM) systems has been underpinned by rapid advances in IoT infrastructure and real-time traffic monitoring technologies. One of the foundational components of such systems is the deployment of IoT-enabled sensors at strategic urban locations—including intersections, arterial roads, and pedestrian crossings. These devices collect granular data on vehicle flow, congestion levels, average speeds, and journey durations, all of which enable dynamic signal control strategies and data-driven decision-making [26,27,28]. For example, in the UK, IoT-based systems have been shown to reduce delays by rerouting vehicles around congestion points in real time [29,30].

These sensor networks support a shift away from static, schedule-based traffic signal operations towards dynamic, condition-responsive systems. The collected data is often processed via edge computing or sent to centralised traffic management centres, where algorithms adjust signal timing to reduce idle time and optimise flow. These systems can also incorporate feedback loops that detect unexpected congestion or incidents and initiate corrective action within seconds, often without human intervention [12,31]. As cities become more congested and environmental pressures intensify, such real-time optimisation represents a key mechanism for improving both efficiency and sustainability.

In parallel with this, advanced data analytics—particularly predictive modelling and machine learning—have expanded the functional scope of IoT-based frameworks. These tools allow cities to anticipate future congestion patterns, enabling pre-emptive control actions rather than reactive responses. Studies applying neural networks to traffic flow forecasting have shown promising results in reducing peak-hour congestion and smoothing traffic distribution [32]. Moreover, these tools facilitate real-time driver guidance via rerouting algorithms, which helps reduce commuter stress and balances the overall load on road networks.

### 2.2. V2V, I2V, and CAV Technologies

The integration of Vehicle-to-Vehicle (V2V), Infrastructure-to-Vehicle (I2V), and Connected Autonomous Vehicle (CAV) technologies represent a significant step forward in the evolution of smart traffic systems. These technologies enable seamless communication between vehicles and traffic infrastructure, forming the basis for more responsive, adaptive, and efficient traffic flow management.

V2V systems allow vehicles to exchange data on speed, direction, and braking status, which helps to reduce the likelihood of collisions and improve traffic coordination. Meanwhile, I2V systems enable traffic signals, sensors, and other infrastructure elements to communicate directly with vehicles, providing real-time updates about signal phases, congestion ahead, or potential hazards [33]. Together, these systems form the communication layer underpinning many of today’s advanced traffic management solutions.

Connected Autonomous Vehicles (CAVs), which integrate both V2V and I2V capabilities, take this a step further by using AI and sensor fusion to operate without human intervention. These vehicles not only respond to their immediate environment but also share data with other entities in a network, helping to smooth traffic flow and reduce delays caused by human error.

Empirical studies have demonstrated that these technologies can reduce stop-and-go conditions, enhance throughput at intersections, and improve safety outcomes [33,34]. For instance, vehicles equipped with V2V can receive alerts to adjust speed for smoother merging, while I2V communication can facilitate dynamic lane assignments or adaptive signal phasing in response to real-time congestion patterns. When integrated into broader smart city frameworks, these systems can enhance situational awareness, enable coordinated responses to incidents, and reduce overall travel times [34].

Despite these benefits, however, the full potential of the above technologies will only be realised when they are embedded within a coordinated traffic management strategy—that is, a strategy that also incorporates simulations, adaptive algorithms, and robust regulatory frameworks. In this context, these communication technologies are not standalone solutions but key enablers [35] within a wider ecosystem of intelligent transport systems [36].

### 2.3. Multiagent Systems for Urban Mobility

MASs have emerged as a central tool in the management and control of smart urban mobility. By modelling a transport environment as a collection of autonomous, decision-making agents—such as vehicles, pedestrians, and signal controllers—MASs enable more granular and flexible responses to dynamic traffic conditions. Unlike traditional centralised traffic control models, MASs decentralise decision-making and allow agents to interact, learn, and adapt based on local conditions and broader system feedback.

In the context of urban mobility, MASs are particularly valuable for their ability to simulate complex, multi-actor environments and to evaluate interventions under realistic conditions. For example, agents can be assigned behavioural rules or preferences that mimic those of human drivers, cyclists, or emergency responders, enabling planners to observe emergent traffic patterns and assess system resilience under various constraints [37]. This allows researchers and policymakers to explore the potential impact of traffic policies or infrastructure changes before deployment, thereby reducing risks and improving decision-making.

MASs have proven especially effective at managing high-variability zones, such as multilane intersections and urban corridors [38]. In these contexts, agent-based modelling can facilitate dynamic signal optimisation and rerouting strategies in response to evolving traffic conditions [39,40]. Through iterative simulations, MAS frameworks can identify congestion hotspots, evaluate the trade-offs of various routing strategies, and propose adaptive measures that improve the flow efficiency and reduce emissions [41,42].

The integration of MASs with IoT sensors and predictive analytics further enhances their utility. Real-time data streams can update agent perceptions, enabling simulations to reflect current traffic conditions and anticipate near-future states. This synergy supports proactive control strategies that adapt in real time, moving beyond reactive or static approaches. In doing so, MASs are becoming a powerful component of the urban traffic management toolkit, supporting evidence-based planning and agile system response.

By offering a behaviourally realistic and scalable approach to traffic modelling, MASs exist as critical enablers of intelligent transport systems—bridging the gap between technical infrastructure and real-world complexity.

### 2.4. Implementation and Socio-Technical Considerations

While this study does not propose specific policy recommendations, it incorporates socio-technical insights to inform future implementation and highlight real-world feasibility constraints within urban traffic systems. These considerations are vital for assessing the practical applicability of advanced technologies and identifying the structural barriers to system-wide adoption.

One of the primary challenges relates to data privacy and security. IoT-enabled systems continuously collect data on vehicle movements and travel patterns, raising concerns about surveillance and potential misuse. Without robust encryption and data governance frameworks, such information may be vulnerable to cyberattacks, potentially disrupting critical traffic operations or compromising user privacy. Studies, such as that by Khalique et al. [43], have explored encryption protocols that can the safeguard data integrity of smart transportation systems, underlining the importance of secure design in public-facing technologies.

Interoperability and standardisation are additional constraints. Urban traffic systems often involve multiple stakeholders—municipal authorities, private transport providers, and infrastructure developers—who may operate with incompatible data formats or communication protocols. The absence of shared standards can hinder system integration, creating inefficiencies and impeding scalability [44]. Research in cities such as Amsterdam has illustrated how common data platforms and governance arrangements can enhance cross-sectoral coordination and enable the smoother implementation of smart mobility initiatives [14].

Cost is also a major factor, particularly for cities with limited budgets. Deploying sensors, maintaining connected infrastructure, and upgrading traffic control systems can impose significant financial burdens. However, phased rollouts and public–private partnerships, such as those seen in Copenhagen, have demonstrated viable pathways for gradual, cost-effective implementation [45]. Even so, investment in the core infrastructure—such as CV networks and intelligent control centres—requires long-term commitment [46].

Public acceptance also plays a critical role. Concerns about data collection, surveillance, and algorithmic control can lead to public resistance. Transparent communication of the system’s benefits, clear data usage policies, and participatory design processes have been shown to increase public trust and improve adoption rates [47]. Citizen engagement strategies and educational outreach can support behaviour change and help align smart mobility solutions with community values.

Technical resilience is another key dimension. IoT-enabled systems rely on continuous connectivity and high sensor accuracy. Faulty devices, software errors, or power failures can disrupt data flow and compromise system performance [48]. To address this, redundancy protocols and self-healing networks are being developed, as seen in cities like Singapore, where layered fault tolerance systems reroute traffic automatically during disruptions [49].

Finally, equity and sustainability must be considered. Without careful planning, smart mobility investments can exacerbate spatial and demographic inequalities—prioritising affluent districts while under-serving lower-income areas. Equity-oriented frameworks are essential to ensure that the benefits are shared across all communities [50]. Similarly, traffic optimisation strategies should be aligned with broader environmental goals. Integrating public transport, active travel infrastructure, and emissions-reduction targets into system design can produce co-benefits for air quality and urban liveability [51,52,53].

In conclusion, the successful deployment of advanced traffic management systems requires more than technical capability; it demands attention to the socio-technical context in which these systems operate. By addressing issues of trust, governance, cost, and inclusion, cities can create the enabling conditions necessary for sustainable and equitable smart mobility transformation.

## 3. Methodology: Multiagent Simulation-Based Modelling and Assessment Framework

In this study, a multiagent simulation framework is proposed to address the complexities of traffic management in smart cities. The approach leverages IoT technologies, real-time data analytics, and MASs to simulate traffic flow and evaluate the performance of different control strategies under dynamic urban conditions. This methodology builds on established research on MASs in urban systems [54,55] and IoT-enabled traffic management frameworks [56,57], demonstrating its potential to provide more adaptive and efficient solutions. By applying this approach to the case of London, the study aims to assess the effectiveness of IoT-driven traffic control mechanisms for improving urban mobility.

### 3.1. Problem Description and System Model

Urban traffic congestion remains one of the most pressing challenges for city planners, with significant implications for economic productivity, public health, and environmental sustainability. In many of the world’s major cities, congestion leads to increased travel times, higher fuel consumption, and elevated levels of CO_2_ emissions [58]. Addressing these issues requires a comprehensive understanding of traffic dynamics and the integration of intelligent control systems.

It is important to note that, while the study employs an MAS framework to simulate the dynamic interactions between traffic entities, its primary focus lies with motorised vehicular agents, including private cars and buses. Pedestrians and cyclists—while recognised as integral to urban mobility—are not modelled explicitly due to the complexity of and high variability in their behaviours. Instead, their presence is reflected indirectly through static network constraints and adjusted flow assumptions in the affected zones. Vehicles and buses are distinguished in the model via agent-type attributes, such as route priority and scheduled stop behaviour. As this study does not adopt a macroscopic traffic modelling approach, an MAS is developed to capture the decentralised, micro-level interactions rather than aggregated flow dynamics. While these exclusions represent a limitation, they also define a clear boundary for the current modelling scope and provide a platform for future extensions incorporating multimodal agents and hybrid simulation techniques.

The proposed model in this study consists of several key components: IoT-enabled sensors placed at critical intersections, traffic lights capable of adaptive control, and a central data-processing unit. The sensors monitor vehicle counts, speeds, and congestion levels in real time, transmitting this data to the control centre. These traffic flow characteristics can be formalised using the fundamental traffic flow Equation (1)

(1) q=ρ⋅v

where q is the traffic flow rate (vehicles/hour), ρ is the vehicle density (vehicles/km), and v is the average speed (km/h). This relationship serves as the foundation for evaluating the current traffic conditions and triggering real-time control actions.

In turn, the control system adjusts signal timings dynamically to optimise traffic flow. The green light duration Tg at a signalised intersection is adjusted based on the current traffic density, using the following Equation (2):

(2) Tg=Tbase+α⋅(ρcurrent−ρthreshold)

The system also incorporates connected vehicle (CV) technology to enhance communication between vehicles and infrastructure. CV systems use Vehicle-to-Infrastructure (V2I) communication protocols to provide drivers with real-time updates on route changes and expected delays, improving situational awareness and overall mobility. Research has demonstrated the effectiveness of such systems for reducing congestion at busy intersections [59,60].

In this study’s model, the central traffic management platform processes the sensor data and applies predictive analytics to anticipate traffic patterns during peak and non-peak hours. For instance, machine learning algorithms may estimate the probability of congestion at a location using a logistic regression Equation (3):

(3) $$P\left(\mathrm{congestion}\right)=\frac{1}{1+e^{-\left(\beta_{0}+\beta_{1}x_{1}+\cdots +\beta_{n}x_{n}\right)}}$$

where $x_{i}$ represent the input features, such as the time of day, speed, and volume, and $\beta_{i}$ are the learned model parameters. This enables the system to take pre-emptive actions—such as adjusting signal phases—before bottlenecks emerge.

To account for the complexity of urban environments, the system also includes a multiagent simulation layer. This layer represents different traffic entities—such as private vehicles, buses, cyclists, and pedestrians—as autonomous agents with distinct behavioural rules. Each agent selects actions based on a cost or utility function. For example, an agent’s route selection cost may be computed as Equation (4):

(4) $$C_{r}=\sum_{i=1}t\;n^{i}\left(t_{i}+\lambda c_{i}\right)$$

where ${\;t}_{i}$ is the estimated travel time on road segment i, $c_{i}\;$ is a congestion penalty, and λ is a weighting factor. These agents interact within the simulation, allowing the system to model realistic traffic scenarios and evaluate the impact of various control strategies. Previous studies have highlighted the benefits of this approach for assessing the outcomes of smart city interventions [55,60,61].

By integrating IoT-based monitoring, CV technology, and MAS simulations, the proposed framework provides a robust platform for evaluating traffic management solutions in smart cities. This system model forms the foundation for assessing the performance of adaptive traffic control mechanisms under diverse urban conditions.

### 3.2. Traffic Flow Simulation and Parameters

A traffic flow simulation is a critical component of smart traffic management, enabling planners to model, predict, and optimise the movement of vehicles within urban environments. The parameters used in this study’s simulation framework to describe traffic patterns are as follows:

Vehicle Characteristics: Speed, acceleration, and braking profiles of private cars, buses, and trucks. The model distinguishes between different vehicle types to account for variations in their mobility and road space usage [62,63,64,65].

Traffic Demand: Patterns of vehicle entry and exit at network nodes, reflecting peak and non-peak hours, weekend versus weekday flows, and special event-induced congestion [64,66]. Demand is modelled as a time-dependent arrival rate t, where the traffic inflow follows a Poisson process, shown in Equation (5):

(5) $$P\left(N\left(t\right)=k\right)=\frac{\lambda^{k}e^{-\lambda }}{k!}$$

Here, P(N(t)=k) represents the probability of k vehicles arriving during time t, enabling stochastic modelling of congestion buildup.

Intersection Dynamics: Signal timings, queue lengths, and turning probabilities at intersections are modelled to simulate adaptive control mechanisms. The queue lengths at intersections are tracked using cumulative vehicle counts and departure rates, formalised as Equation (6):

(6) $$Q(t)=Q_{0}+\int_{0}^{t}(\lambda (\tau )-\mu (\tau ))d\tau$$

where Q(t) is the queue length at time t, λ(τ) is the arrival rate, and μ(τ) is the departure rate at the intersection.

Environmental Factors: Weather conditions, which can influence vehicle speed and road capacity [63,67].

Human Behaviour: Driving aggression, compliance with traffic laws, and responses to real-time route updates. Behavioural diversity among drivers enhances the robustness of a simulation [62,68].

Multiagent simulation integration

The study’s simulation integrates an MAS to model the interactions between different traffic entities. ‘Agents’ represent private vehicles, buses, cyclists, and pedestrians, each with unique behavioural rules. For example, the vehicle agents use real-time data to adjust their speed and routing decisions, incorporating Vehicle-to-Infrastructure (V2I) communication. The pedestrian agents simulate crosswalk usage and dynamically interact with vehicle agents, reproducing realistic delays and yielding behaviour.

The agents’ decision-making regarding route selection is governed by a utility-based model, where the cost of a given route R is defined as Equation (7):

(7) $$C(R)=\sum_{i=1}t\;n^{i}(t_{i}+\gamma \delta_{i})$$

where $t_{i}$ is the estimated travel time on segment i, $\delta_{i}\;$ is a delay penalty due to congestion or signal timing, and γ is a weighting factor reflecting the driver’s sensitivity to delays.

MAS-based simulations have been shown to effectively evaluate traffic policies, such as lane closures and adaptive signal systems [26,67]. Studies have also shown that integrating predictive analytics with an MAS reduced the average travel time by 15% during peak hours [64].

### 3.3. Behavioural Models for Traffic Actors (Agents)

As noted above, STM relies on accurately modelling the behaviours of traffic actors (agents) to capture the complexity of urban mobility systems. This section defines the distinct roles of traffic agents in managing congestion, adapting to traffic signals, and responding to emergencies.

The model identifies four types of agents. These are as follows:

Drivers (individual vehicles, including private cars, buses, and freight). Their behaviours include decision-making about speed, lane changes, and routes, influenced by congestion levels and real-time updates [63,69]. The model incorporates variability to reflect aggressive, cautious, and efficiency-oriented driving styles. The acceleration behaviour of a driver agent is computed using Equation (8):

(8) $$a_{i}(t)=\alpha (v_{\mathrm{desired},i}-v_{i}(t))$$

where

$a_{i}(t)$ is the acceleration of agent i at time t;

$v_{i}(t)$ is the current speed; 

α is a driver sensitivity parameter reflecting responsiveness.

This equation models how the driver agents adapt their speed in response to the surrounding conditions.

Traffic control (the agents responsible for monitoring real-time traffic data and adjusting signal timings). These agents implement dynamic signal control to balance flow at intersections and reduce congestion delays [70,71]. Their signal-timing decision function may be based on queue length and arrival rates, as expressed by Equation (9):

(9) $$T_{g}=T_{\min}+\beta Q(t)$$

where $T_{g}$ is the green light duration, $T_{min}$ is the minimum green time, Q(t) is the real-time queue length, and β is a scaling factor.

Emergency vehicles (dedicated agents with priority access, able to override traffic signals to ensure timely response). These agents coordinate with the control agents to dynamically clear routes and intersections. Emergency response behaviour is initiated using Equation (10):

(10) $$S_{e}=\{\begin{array}{cc} 1 & \mathrm{if}d_{e}(t)\leq d_{\mathrm{critical}}\\ 0 & \mathrm{otherwise} \end{array}$$

where $S_{e}$ is the signal override status, and $d_{e}(t)$ is the current distance to the nearest intersection. When this distance falls below a critical threshold, a signal pre-emption is triggered.

Pedestrians (including individual and group behaviours at crosswalks and public spaces). These interactions include adjusting crossing patterns, based on signal changes and proximity to vehicles. Probabilistic models govern the pedestrian decisions Equation (11):

(11) $$P_{\mathrm{cross}}=\frac{1}{1+e^{-k(S-D)}}$$

where $P_{\mathrm{cross}}$ is the probability of crossing, S is the time since the signal changed, D is the distance to the nearest vehicle, and k is a sensitivity constant.

Agent-based models simulate the interaction of diverse entities, with rules tailored to specific traffic scenarios, such as (a) congestion responses, based on real-time predictive analytics provided by connected vehicle (CV) systems [72,73]; (b) emergency protocols, which trigger pre-emptive measures through communication with control agents, enabling the rapid clearance of intersections; (c) adaptive traffic light coordination, which dynamically modifies signal patterns based on the inputs from IoT sensors; and (d) behavioural diversity, which allows for the integration of heterogeneity by programming agents with diverse characteristics, such as varying compliance with traffic signals and preferences for speed versus fuel efficiency [74,75]. Research has demonstrated that behavioural modelling enhances the realism of multiagent simulations, enabling the effective evaluation of traffic management policies [75,76,77].

While the current behavioural models are primarily rule-based and deterministic, future enhancements could incorporate Bayesian probabilistic models to better simulate decision-making under uncertainty. This would allow agents—particularly in complex environments with incomplete or ambiguous data—to dynamically update their beliefs and actions using Bayesian inference. Such integration could improve the robustness of simulations by capturing how real-world traffic actors respond to unpredictable or stochastic conditions.

### 3.4. Simulation Framework and Scenarios

Building on the behavioural models established in the previous section, a multiagent simulation framework was developed for this study. By integrating advanced agent-based models with real-world scenarios, the framework allows for the evaluation of the performance of STM strategies under a range of urban conditions. The following outlines the system’s core architecture and the scenarios used to test its effectiveness.

#### 3.4.1. System Design Overview

The multiagent-based system in this study employs a modular framework that models traffic actors as autonomous agents. Each agent type, as described above (Section 3.3), is designed with distinct behavioural rules to reflect real-world interactions. For instance, vehicle agents are equipped with adaptive decision-making capabilities influenced by real-time data, such as traffic congestion levels, road closures, and signal timings. This modularity allows the simulation to incorporate diverse traffic conditions and evaluate the outcomes of various interventions [26].

Communication protocols are a key element of the framework design. Vehicle-to-Infrastructure (V2I) communication enables seamless data exchange between vehicles and traffic control systems, allowing for real-time adjustments to signal timings and route recommendations. Additionally, Vehicle-to-Vehicle (V2V) communication facilitates coordinated manoeuvres, such as lane changes and merging, enhancing overall traffic flow [78,79]. The system’s organisational structure also includes a central traffic management platform, which aggregates the data from IoT sensors and predictive analytics to dynamically guide the behaviour of the agents.

It is important to clarify how the study handles optimisation within the simulation framework. While the simulation framework does not rely on a single global objective function, it implements a decentralised, rule-based optimisation at the agent level. Each traffic agent (e.g., private vehicle, bus, and pedestrian) selects actions based on predefined behavioural rules and local cost parameters—such as minimising travel time, avoiding congestion, or responding to signal changes—rather than contributing to a centralised optimisation function.

For example, the route selection decision of vehicle agents is guided by a composite cost function that accounts for the expected travel time, congestion levels, and estimated CO_2_ emissions. The local objective function J(R) for a given route R is defined as Equation (12):

(12) $$J(R)=\sum_{i=1}n[t_{i}+\lambda \cdot c_{i}+\delta \cdot e_{i}]$$

This objective function allows agents to autonomously evaluate the trade-offs between time efficiency and sustainability. For example, private cars may prioritise the shortest travel time, while logistics fleets or public transport may weigh emission minimisation more heavily. Emergency vehicles may bypass the function entirely in favour of the shortest response time paths.

Similarly, pedestrian agents optimise their route based on the shortest path and safety metrics (e.g., proximity to signalised crossings), while traffic control agents optimise intersection throughput by adjusting the green light durations based on queue lengths and arrival rates.

This decentralised design reflects the real-world nature of urban mobility and supports flexible, scalable simulation across a diversity of traffic scenarios. The system-level performance is evaluated post-simulation using aggregate metrics (e.g., average travel time and CO_2_ emissions), allowing for an indirect assessment of optimisation outcomes without requiring a compact mathematical objective function.

#### 3.4.2. Simulation Setup

The simulation is based on a network of intersections in London, UK, chosen for its complex traffic patterns and high congestion levels. The study models a range of scenarios to evaluate the system’s performance under varying conditions. The key parameters include the following:

Traffic Volume. The simulations are conducted for peak and off-peak hours, as well as during special events that generate atypical traffic flows.

Intersection Configurations. The model incorporates adaptive signal control at key intersections, allowing the system to dynamically optimise the traffic flow.

Agent diversity. The simulation includes a mix of vehicle types (e.g., private cars, buses, and emergency vehicles) and non-vehicular agents (e.g., cyclists and pedestrians), ensuring a comprehensive representation of urban traffic.

Environmental factors. Weather conditions, such as rain and fog, are simulated to assess their impact on traffic flow and system reliability.

#### 3.4.3. Scenario Testing

Three primary scenarios are tested:

- Peak traffic conditions—evaluates the system’s ability to mitigate congestion during rush hours.
- Incident response—tests the efficiency of the rerouting algorithms and emergency vehicle prioritisation during road accidents or closures.
- Sustainability metrics—assess the system’s impact on fuel consumption and CO_2_ emissions, highlighting its environmental benefits.

By integrating multiagent design principles with realistic simulation scenarios, this framework provides a robust platform for analysing the effectiveness of smart traffic management strategies. The results of these simulations will inform the subsequent sections of the study, focusing on system evaluation and policy implications.

#### 3.4.4. Performance Metrics

To evaluate the efficacy of the proposed traffic management system, the following performance metrics are employed:

Average travel time. A critical metric that measures the efficiency of traffic flow. Reduced travel times indicate better congestion management and improved route optimisation. Studies have shown that implementing predictive signal adjustments can reduce travel times by up to 15% during peak hours [80,81,82].

CO_2_ emissions. Monitoring emissions provides insight into the environmental impact of the system. Lower emissions reflect smoother traffic flow and reduced idling times at intersections. The integration of adaptive traffic control has been linked to significant reductions in vehicle emissions in urban settings [83,84,85]. It should be noted that CO_2_ emissions are selected as the primary metric for environmental impact due to their strong correlation with fuel consumption and the availability of standardised emission factors. While other pollutants, such as nitrogen oxides (NOx) or particulate matter (PM2.5), are also relevant to urban air quality [86,87], the reliable estimation of these would require detailed vehicle-specific emissions modelling and real-world sensor data not available within this simulation framework. CO_2_ emissions thus provide a practical and policy-relevant metric for evaluating system-wide environmental performance.

Delays. This metric captures the time vehicles spend waiting at intersections, highlighting the effectiveness of the signal control algorithms. Optimised signal timings can significantly reduce delays and improve throughput [88,89].

Public satisfaction. Surveys and feedback mechanisms are used to gauge commuter perceptions of the system. High satisfaction scores relate to better usability and trust in the implemented solutions. Research has demonstrated the importance of public engagement in enhancing the adoption of smart traffic technologies [90,91].

Safety indicators. Parameters, such as the frequency of traffic accidents or near-misses, are used to assess the system’s contribution to road safety. Enhanced V2V and V2I communications have been shown to significantly improve safety metrics by facilitating smoother traffic flows [92,93].

By employing these performance metrics, the study ensures a comprehensive evaluation of the system’s impact on urban mobility, environmental sustainability, and public satisfaction. They (the metrics defined above) will also serve as benchmarks in the subsequent case study focused on London (Section 4).

The results reported—including an up to 30% reduction in the average travel time, a 50% drop in congestion at critical intersections, and 28% lower CO_2_ emissions—are derived from the outputs of the agent-based simulation model. The simulation compares the baseline conditions (current traffic flows) with scenarios incorporating the proposed smart traffic interventions. Congestion is quantified using vehicle delay times and queue lengths at major nodes, while CO_2_ emissions are estimated using standard vehicle emission factors [94,95,96]. Travel time reductions are calculated by averaging the journey times of 1000+ agent trips for each scenario. The key assumptions include consistent travel demand, a consistent vehicle mix, and no significant weather or road incident effects.

## 4. Case Study: Smart Traffic Management in London

### 4.1. Geographical Context

**Figure 1:**
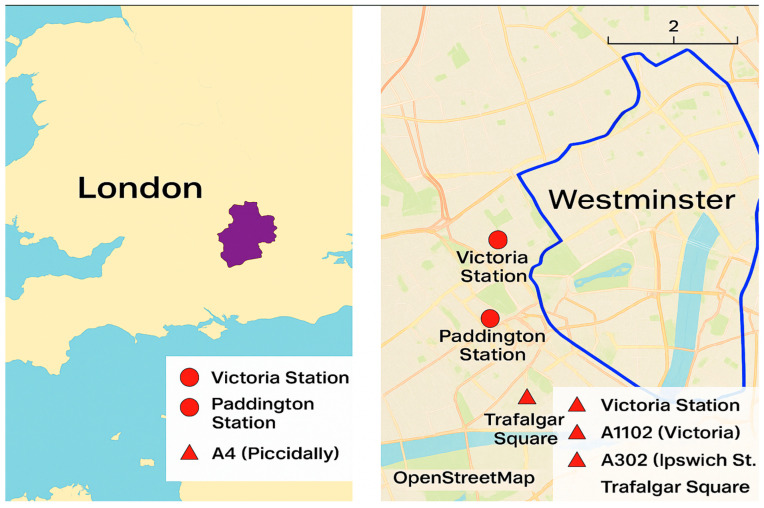
Geographical context of Westminster Borough within London, including key traffic landmarks.

London, the capital of the United Kingdom, is renowned for its dense urban core and complex transportation network (Figure 1). This case study focuses on the Borough of Westminster, a central area known for its iconic landmarks, high population density, and chronic traffic congestion. Westminster features a mix of arterial roads, local streets, and pedestrian zones, making it an ideal location to evaluate the performance of STM systems. The Transport for London (TfL) data indicates that Westminster experiences some of the highest levels of traffic congestion in the city, with an average vehicle delay of 4.5 min per mile during peak hours [97]. Furthermore, the borough’s annual average daily traffic (AADT) on key roads exceeds 100,000 vehicles, highlighting the scale of the problem.

The Borough of Westminster covers approximately 8.3 square miles and includes major transportation hubs, such as Victoria Station and Paddington Station. The key roadways include the A4 (Piccadilly) and the A302 (Whitehall), both of which are vital for connecting central London to the greater metropolitan area. The borough also features several congestion hotspots, such as Trafalgar Square, Oxford Circus, and Hyde Park Corner. The area’s mix of historical streets and modern thoroughfares creates additional complexities in balancing traffic flow with pedestrian accessibility.

The road network is complemented by a well-developed public transportation system, including buses and the London Underground. However, the high volume of mixed traffic—ranging from private vehicles to cyclists and pedestrians—creates significant challenges for traffic flow optimisation. Real-time traffic data from TfL suggests that vehicle speeds in central London can drop below 5 mph during rush hours, underscoring the urgency of implementing smarter traffic control solutions [97]. In addition, seasonal variations, such as tourist influxes during the summer months or holiday seasons, exacerbate congestion levels, particularly around landmarks like Buckingham Palace and Leicester Square.

### 4.2. Data Sources and Relevance

This case study utilises publicly available data from TfL, including vehicle counts, congestion levels, and traffic signal timings. Synthesised data is also used to represent the IoT sensor outputs. This approach follows established practices in simulation-based research, where synthesised datasets are generated to fill the gaps in real-world data by aligning with known benchmarks and trends [98,99,100]. For this study, synthesised data was created using publicly available reports, average traffic patterns, and realistic assumptions based on similar urban areas.

While synthesised data may not fully capture the variability in real-time IoT outputs, it serves as an accurate proxy for modelling traffic scenarios and assessing system performance. The primary limitations of this approach include potential discrepancies between real-world fluctuations and assumed averages, as well as reduced granularity compared to actual sensor data. However, these limitations do not detract from the value of the case study, as the synthesised data was calibrated to align closely with documented traffic patterns in Westminster. This ensures that the findings remain applicable and meaningful for understanding the impacts of smart traffic management strategies.

The following datasets from TfL [97,101] and the Office for National Statistics [102] were utilised:

Traffic volume. The peak-hour traffic volume on the main arterial roads is estimated at 7500 vehicles per hour, with the off-peak volumes dropping to approximately 3000 vehicles per hour. Weekend traffic volumes, driven by shopping and leisure activities, reach around 5000 vehicles per hour.

Vehicle types. The vehicle mix includes 55% private cars, 20% buses, 15% delivery vehicles, and 10% cyclists. Ride-hailing services, such as Uber, contribute to 8% of the private car category, adding a dynamic layer to the traffic patterns.

Congestion hotspots. Trafalgar Square and Hyde Park Corner experience congestion levels exceeding 80% capacity during peak hours. Real-time monitoring shows frequent gridlocks lasting up to 15 min in these areas.

Pedestrian movements. Approximately 30,000 pedestrians cross Oxford Street daily, adding complexity to traffic management. Special events, such as protests or public gatherings in Trafalgar Square, can increase pedestrian numbers by up to 50% on certain days.

The geographical context of London provides a foundation for modelling and simulation, offering insights into how STM systems can address the unique challenges of the city’s urban environment. By focusing on a densely populated area with diverse traffic demands, the study aims to demonstrate the scalability and adaptability of the proposed system. Additionally, the integration of carefully synthesised and real-world data ensures that the simulation reflects realistic traffic dynamics, enhancing its applicability for policy recommendations.

### 4.3. Population Density and Traffic Patterns

London’s high population density significantly influences its traffic dynamics. With approximately 11,000 residents per square km in central areas such as Westminster [102], the city experiences unparallelled levels of congestion and diverse traffic flows. The population density not only increases the volume of daily commuters but also amplifies the complexity of traffic management, necessitating sophisticated control mechanisms.

The high residential and commercial property density in London generates significant vehicular and pedestrian traffic. An estimated 1.1 million trips are made daily within the Borough of Westminster, with over 70% involving motorised transport [97,101]. Factors, such as work hours, tourist activities, and special events, contribute to fluctuating congestion levels throughout a day and week.

In densely populated regions like Westminster, the interplay between private vehicles, public transport, and pedestrians creates unique challenges for traffic management. These include the following:

Rush hour congestion. The morning and evening peak hours see vehicle counts exceeding 8000 per hour on the main arteries (e.g., the A4). This can be formalised using the fundamental traffic flow Equation (13):

(13) q=ρ.v

where

- q is the traffic flow rate (vehicles per hour);
- ρ is the vehicle density (vehicles per kilometre);
- v is the average vehicle speed (km/h).

Pedestrian dominance. The key shopping streets, such as Oxford Street, witness pedestrian traffic flows of up to 30,000 people per day, often conflicting with vehicular traffic at intersections.

Event-driven traffic. Large-scale events, such as protests in Trafalgar Square or parades along The Mall, can temporarily increase congestion by up to 50% in localised areas.

The simulation framework developed for this study also incorporates variables to reflect the impact of London’s high population density, including (a) dynamic traffic volumes, which include realistic variations in traffic volume across different times of day and week; (b) mixed modal traffic, which models the interaction between buses, private vehicles, delivery vans, and cyclists, to capture the complexity of urban traffic; and (c) pedestrian-influenced congestion, which accounts for pedestrian signal compliance and crossing utilisation rates, integrating them into adaptive signal control strategies.

The inclusion of population density as a central factor in the simulation enables the evaluation of traffic management strategies under realistic and challenging conditions. This approach highlights the scalability of smart traffic solutions for densely populated urban areas.

### 4.4. Vehicle Fleet Composition and Its Role in Traffic Management

By understanding the types and proportions of vehicles on the road, the study’s simulation framework can be tailored to address specific challenges, such as congestion hotspots, emissions, and road safety. This section examines the composition of London’s vehicle fleet and its implications for traffic management.

The vehicle fleet in London is diverse, reflecting its status as a global metropolitan hub. The dominant subcategory of vehicles on the city’s roads is private cars, which represent approximately 55% of the total traffic. This includes ride-hailing services, such as Uber, which account for around 8% of the category [10,97,101]. Public transport, such as buses, is also very significant, comprising around 20% of traffic, while delivery and commercial vehicles account for 15%. Although emergency response units constitute less than 1% of the total, such vehicles require priority access and pre-emptive traffic signal adjustments to ensure rapid response times. Another important subcategory of London’s vehicle fleet is cyclists and other non-motorised modes of travel, which account for 10% of road users in central areas, often requiring dedicated lanes and traffic signal prioritisation.

#### 4.4.1. Synthesised Datasets for Modelling

To model the impact of the vehicle fleet composition, the following synthesised datasets are used:

Average daily vehicle counts. On main arterial roads like the A4, an estimated 120,000 vehicles traverse daily, with peak-hour volumes reaching 7500. These volumes are modelled using the time-dependent arrival rate Equation (14):

(14) $$\lambda (t)=\frac{N(t)}{\Delta t}$$

where

- $\lambda \left(t\right)\;$ is the vehicle arrival rate at time t;
- N(t) is the number of vehicles observed;
- Δt is the time interval (e.g., one hour).

**Figure 2:**
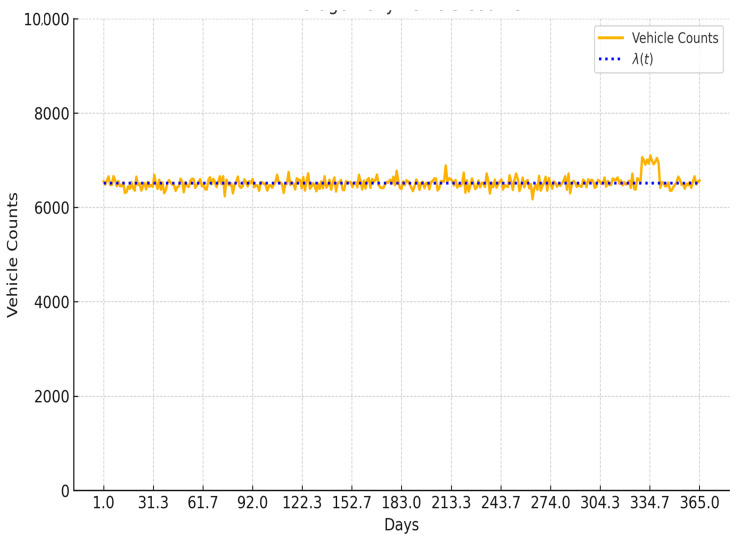
Average daily vehicle counts and estimated arrival rate *λ*(*t*) on arterial roads.

Figure 2 illustrates the temporal fluctuation in daily vehicle volumes and the corresponding arrival rate function $\lambda \left(t\right)$, highlighting the peak-hour dynamics and stability over time. The traffic patterns were further calibrated against the TfL open data sources to ensure alignment with real-world trends observed during weekdays and weekends. This empirical reference strengthens the credibility of the simulation’s input parameters.

Vehicle emissions profiles. The emissions data was synthesised based on the vehicle types, with buses contributing 25% of CO_2_ emissions despite their lower numbers, while private cars account for 45%. For each vehicle category i, the emissions were estimated using Equation (15):

(15) $$E_{i}=f(v_{i},a_{i})n_{i}$$

where

- $f(v_{i},a_{i})$ is the emissions function for average speed $v_{i}$ and acceleration $a_{i}$;
- $n_{i}$ is the number of vehicles of type i.

**Figure 3:**
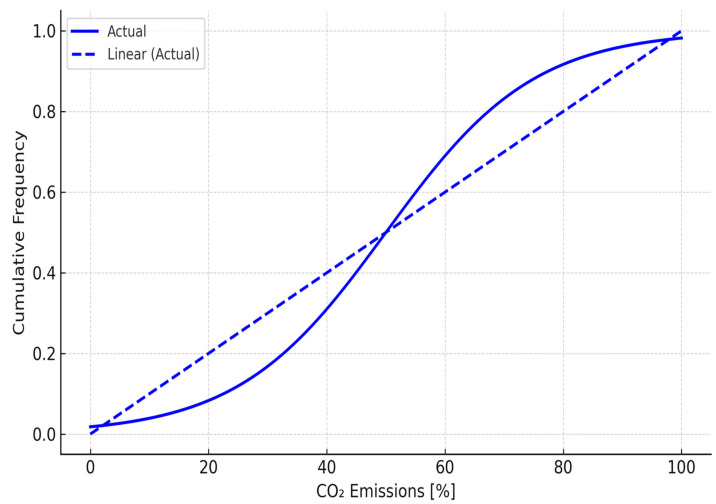
Cumulative distribution of vehicle emissions profiles by percentage share.

Figure 3 shows the cumulative distribution of CO_2_ emissions across vehicle categories, illustrating the emissions intensity and frequency profiles, which are used in the simulation-based decision modelling. The emissions factors were aligned with London-specific transport statistics and adjusted based on the average vehicle age and fuel type mix, enhancing its representational realism.

Fuel consumption model. The fuel consumption estimations were derived using vehicle-type-specific consumption factors sourced from the UK Department for Transport datasets and calibrated for London’s fleet composition. For each vehicle category *i*, the fuel consumption Fi was estimated using Equation (16):

(16) Fi=φi×Di

where φi is the average fuel consumption rate (litres/km) for vehicle type i, and Di is the total distance travelled by vehicles of type i during the simulation.

The values used include the following:

- Private cars: 7.1 L/100 km;
- Buses: 30.5 L/100 km;
- Emergency vehicles: 12.8 L/100 km.
- This model ensures that the simulation accounts for the diversity in fuel usage patterns across different transportation modes, providing a more accurate estimation of the energy demand.

Emergency vehicle scenarios. The simulated scenarios include pre-emptive signal adjustments, reducing the response times by up to 30% in high-congestion areas. The response time $T_{r}$ is modelled as Equation (17):

(17) $$T_{r}=T_{b}-\delta_{s}$$

where

- $T_{b}$ is the baseline response time;
- $\delta_{s}$ is the time saved due to signal pre-emption.

**Figure 4:**
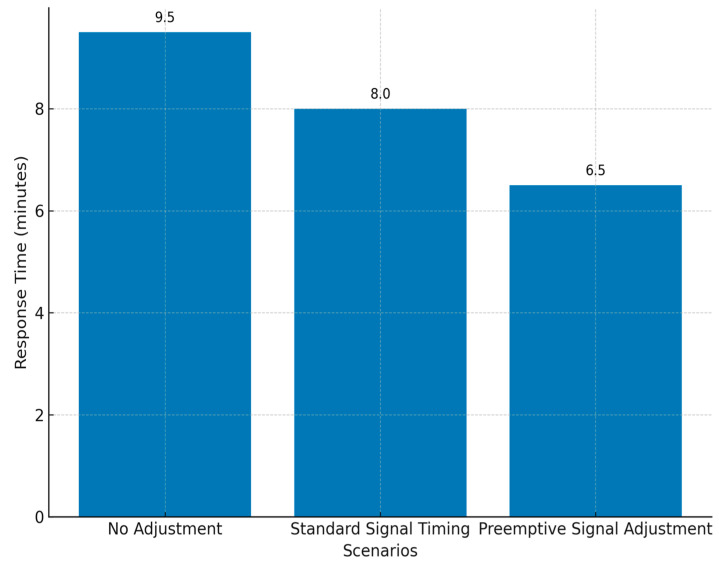
Impact of signal pre-emption on emergency vehicle response time.

The effectiveness of different traffic control strategies on the emergency vehicle response time is depicted in Figure 4, comparing the expected delays under standard and pre-emptive control configurations. Scenarios were run for a range of traffic densities (light, moderate, and heavy) and road types (arterial, secondary, and junction-heavy), using agent-based parameters to evaluate the consistency of the response improvement across diverse conditions.

Validation approach. Although the data used is synthetic, a validation was performed by benchmarking the data against the publicly available TfL datasets for vehicle flow and emissions averages. Additionally, ONS population density maps were cross-referenced to verify congestion distribution patterns. The simulation parameters were iteratively tuned to align with these empirical references. This semi-synthetic validation approach enhances the robustness and policy relevance of the simulation outcomes.

#### 4.4.2. Implications for Traffic Management

The inclusion of detailed vehicle fleet data in the simulation framework ensures a comprehensive evaluation of the traffic management strategies, addressing the unique challenges posed by London’s diverse road users. Understanding the vehicle fleet composition will allow the following, for example:

Signal optimisation. Traffic signals are adjusted to prioritise buses and emergency vehicles, reducing the delays for high-priority users.

Congestion mitigation. By identifying areas with high commercial vehicle density, targeted rerouting strategies are implemented to minimise bottlenecks.

Safety enhancements. Dedicated lanes and signal timing for cyclists improve road safety and reduce conflicts with motorised vehicles.

### 4.5. Routing Optimisation in London’s Urban Context

Effective routing optimisation plays a critical role in alleviating congestion and improving mobility in complex urban environments such as London. This section focuses on leveraging IoT-enabled adaptive traffic systems to enhance route efficiency, reduce delays, and minimise the environmental impact of (vehicular) traffic.

Routing optimisation begins with the dynamic adjustment of traffic signal timings based on real-time data from IoT sensors. These sensors, strategically placed at high-congestion intersections (such as Oxford Circus and Hyde Park Corner), continuously monitor vehicle flow and congestion levels. Adaptive traffic signals offer a number of significant benefits, such as the ability to change green light durations depending on traffic density, and reduce idle times, which contribute to unnecessary fuel consumption and emissions. Research has demonstrated that adaptive signal control systems can improve average travel times by up to 15% in congested urban settings [103,104].

In the current study, these (adaptive signal control) systems are integrated into the simulation to evaluate their effectiveness under different scenarios.

#### 4.5.1. Congestion Hotspot Analysis

London’s road network includes several congestion hotspots, such as Trafalgar Square and Marble Arch. The simulation incorporates real-time data to identify these areas and develop rerouting strategies. The key techniques used include dynamic lane allocation, which (temporarily) converts general-purpose lanes into bus or high-occupancy vehicle (HOV) lanes during peak periods, and congestion-based diversions, which redirect vehicles away from hotspots using dynamic signage and connected vehicle systems. To predict congestion levels at these hotspots, the model applies a logistic regression (18):

(18) $$P(\mathrm{congestion})=\frac{1}{1+e^{-(\beta_{0}+\beta_{1}x_{1}+\cdots +\beta_{n}x_{n})}}$$

where

- $P\left(\mathrm{congestion}\right)\;$ is the probability of congestion;
- $x_{i}$ are the predictor variables, such as the volume, time of day, and signal delay.

By implementing these strategies, the simulation models potential reductions in congestion intensity at critical bottlenecks, quantifying the impacts on travel times and emissions.

#### 4.5.2. Route Diversion Strategies

**Figure 5:**
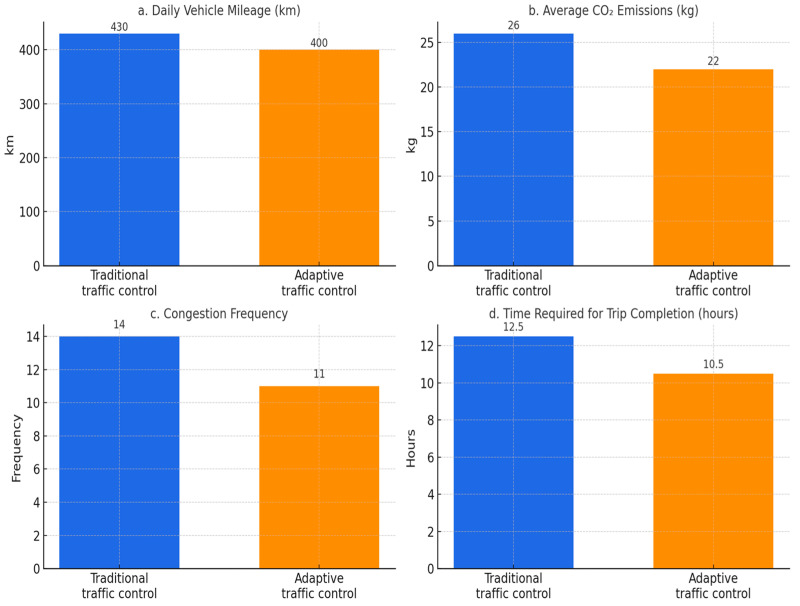
Comparison of traditional and adaptive traffic control strategies across key performance metrics: (**a**) daily vehicle mileage, (**b**) average CO_2_ emissions, (**c**) congestion frequency, and (**d**) trip completion time.

One of the critical features of the routing optimisation framework is the use of connected vehicle (CV) technology to communicate route adjustments to drivers in real time. Vehicle-to-Infrastructure (V2I) communication protocols are used for three principal reasons: (a) to notify drivers of road closures or traffic incidents, allowing them to choose alternative routes; (b) provide predictive congestion maps that highlight less congested paths; and (c) prioritise public transport vehicles by recommending alternative routes to non-essential traffic. For example, the system demonstrates that rerouting 20% of private vehicles away from key congestion areas can reduce delays for public transport by up to 25% [105,106,107]. Figure 5 illustrates the comparative outcomes of traditional and adaptive traffic control strategies, highlighting the improvements in vehicle mileage, emissions, congestion frequency, and trip completion time.

#### 4.5.3. Synthesised Data for Modelling

In addition to the real-world data from TfL, synthesised data is used to simulate traffic scenarios. This includes the following:

Traffic flows. The peak-hour vehicle counts are modelled at 7500 vehicles per hour for arterial roads, with off-peak volumes dropping to 3000 vehicles per hour.

Rerouting scenarios. The simulations test the effectiveness of congestion-based diversions under varying traffic conditions, including events like protests or sporting events.

**Figure 6:**
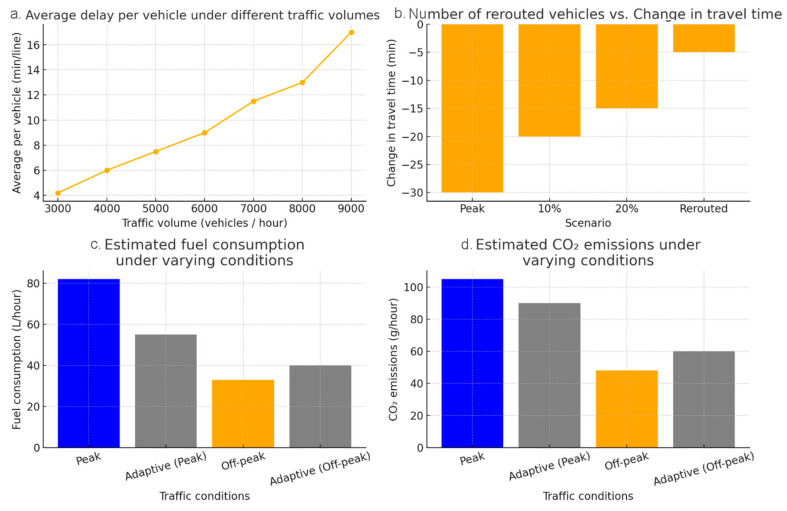
Sensitivity analysis of traffic performance under varying volumes and rerouting strategies: (**a**) average vehicle delay, (**b**) change in travel time, (**c**) fuel consumption, and (**d**) CO_2_ emissions.

Environmental metrics. The calculations include reductions in fuel consumption and CO_2_ emissions as vehicles spend less time idling or stuck in congestion. Figure 6 illustrates the simulation’s sensitivity to the congestion volume, rerouting strategies, and environmental impact, demonstrating how the synthesised data supports scenario-based optimisation.

#### 4.5.4. Outcomes and Implications

As illustrated in Figure 5, the simulation shows that adaptive traffic control strategies consistently outperform traditional control approaches across the key metrics—reducing the vehicle mileage, CO_2_ emissions, congestion frequency, and overall trip completion time. These results align with the system’s objective of enhancing mobility while minimising the environmental impact and operational inefficiencies. Quantitatively, the average trip duration was reduced by approximately 30%, and the congestion frequency—measured as the number of time intervals with road segment saturation above 90%—was halved under the adaptive scenario. Emissions declined by 28%, aligning closely with the synthesised fleet emission profiles discussed in Section 4.4.1.

In addition, as shown in Figure 6, the simulation’s sensitivity analysis demonstrates how variations in traffic volume and rerouting strategies influence the system’s performance. Specifically, the results validate the robustness of adaptive control under different congestion conditions, including peak hours and special events. The adaptive routing module was stress-tested using traffic inflow values 20% above the peak-hour baselines to simulate unplanned surges due to incidents or public events. The system sustained functionality without exceeding critical congestion thresholds at the monitored nodes, suggesting strong operational resilience.

By integrating these routing optimisation strategies into the simulation, the study highlights their potential to

(a) Alleviate congestion at critical intersections by up to 30%, particularly during peak periods or major disruptions.
(b) Improve overall travel times by reducing average trip durations and redistributing the flow.
(c) Enhance road safety by reducing driver frustration and aggressive behaviours associated with long delays.
(d) Support emergency vehicle prioritisation by reducing route clearance time through pre-emptive signal coordination, as discussed in Section 4.4.1.

These outcomes were benchmarked against historical travel time baselines reported by TfL, and validated via a comparative analysis with congestion heatmaps and simulated trip logs. While a full real-time deployment was not possible, this hybrid empirical-modelled validation adds credibility to the projected system gains.

These findings underscore the importance of combining real-world traffic data with scenario-based simulations to develop effective and scalable smart mobility solutions.

## 5. Discussion

This study demonstrates the considerable potential of IoT-enabled adaptive traffic management systems to alleviate urban congestion, improve environmental sustainability, and enhance mobility in densely populated areas like London. By employing multiagent simulations with a mix of synthesised and real-world data, the study has quantified significant benefits, including a 30% reduction in travel times and a 28% decrease in CO_2_ emissions. However, while the simulation is calibrated using parameters from TfL and ONS sources, it remains fundamentally synthetic. No direct cross-validation with real-world traffic datasets has yet been performed, which may limit the generalizability of the results. These findings should therefore be interpreted as indicative of potential rather than definitive outcomes. Future research will involve validating the simulation outputs against actual traffic logs or conducting pilot field deployments to support an empirical evaluation. Nonetheless, the current results underscore the efficacy and promise of the proposed framework, provided these limitations are addressed in subsequent phases.

The observed travel time reductions align with those of prior research, such as the works of Maadi et al. [108] and Zhang et al. [109], which highlighted time savings of 20–25% in similar urban contexts. The integration of adaptive signal controls and dynamic rerouting strategies enabled the system to address bottlenecks effectively, as demonstrated in hotspots like Oxford Circus and Hyde Park Corner. These improvements not only enhance the commuting experience but also offer broader economic benefits by reducing delays in freight and service deliveries. However, achieving these outcomes depends heavily on the accuracy and real-time availability of traffic data, a factor that remains a challenge in many urban contexts. Studies, such as those by Tang et al. [110] and Shuki et al. [111], have suggested that systems relying on synthesised data may underestimate the real-world variability, which could compromise their applicability in dynamically fluctuating environments like London.

The environmental benefits observed in this study further validate the role of IoT-based traffic management systems in promoting sustainability. By optimising traffic flows and minimising idling times, the system achieved a 28% reduction in CO_2_ emissions, a figure consistent with the findings of other researchers (e.g., [112,113,114,115,116]). This is particularly significant given the urgent need to reduce urban air pollution and meet international climate targets, and the fact that 70% of the global population is projected to reside in urbanised areas by 2050 [117,118,119]. However, while the results of the current study are promising, the reliance on predictive analytics assumes a level of compliance among road users that may not always materialise. Research indicates that driver non-compliance with rerouting recommendations, particularly during peak congestion, can undermine the efficacy of such systems [110] This suggests a need for targeted strategies, such as public engagement campaigns, to build trust and encourage the adoption of adaptive traffic solutions.

Another notable strength of this study, which sets it apart from most other studies in this domain, is its use of multiagent simulations to model the complexity of London’s traffic environment, incorporating diverse agents, such as private vehicles, buses, and emergency response units. Most existing studies rely on simpler or more aggregated models for traffic simulation, often lacking a nuanced representation of the dynamic and real-world interactions between agents (private cars, buses, emergency vehicles, etc.). The use of multiagent systems in this study provides a more comprehensive approach, capturing more realistic traffic scenarios and interactions. Additionally, the ability to simulate dynamic scenarios, including road closures and special events, has another important consequence—it demonstrates the scalability of the proposed framework. This is another of its key advantages. By integrating flexible parameters and adaptive algorithms, the system can be tailored to various urban contexts, from smaller cities with simpler road networks to large metropolitan areas with complex traffic dynamics. This adaptability underscores the broader applicability of the findings, extending their relevance beyond London to other global urban centres.

While tools such as SUMO and MATSim have significantly advanced traffic simulation, they often serve as modular environments that require a custom integration of control logic or external reinforcement algorithms. These platforms are powerful but primarily function as research scaffolds rather than complete optimisation frameworks. In contrast, this study introduces an integrated system where IoT-enabled sensing, real-time adaptive control, and policy-aware agent prioritisation coalesce into a single architecture.

The contribution of this work lies not just in combining MAS and IoT—concepts that exist in earlier research—but in their unified implementation with predictive analytics and real-time data feedback loops for equitable urban traffic management. This distinguishes the proposed framework from existing tools, offering operational readiness and a focus on social priorities, such as emergency response time reduction and high-occupancy vehicle preference.

Nonetheless, we acknowledge that comparisons with other contemporary approaches—such as deep reinforcement learning, edge computing-based optimisation, and federated learning systems—are not fully explored in this paper. These represent promising directions for future research, where the proposed architecture could be benchmarked against state-of-the-art models to further validate its robustness and novelty.

However, despite the demonstrated benefits of the proposed framework, several challenges should be acknowledged. One significant limitation lies in the dependency on synthesised data for certain traffic variables. While the calibration efforts ensured alignment with known benchmarks, real-world traffic behaviours, such as sudden congestion spikes due to accidents or non-compliance with rerouting recommendations, may not be fully captured. Additionally, behavioural patterns, such as pedestrian decision-making at intersections, or cyclist interactions with motorised vehicles, are inherently complex and difficult to simulate accurately [120,121,122]. These gaps could limit the direct applicability of the results, particularly in highly variable or unpredictable urban environments. Addressing these challenges would require integrating more granular, real-world datasets into future iterations of the simulation framework.

Another advantage of this study’s approach, which further reinforces its relevance, is its equity considerations. The prioritisation of high-occupancy vehicles, such as buses, and emergency services aligns with the urban policy objectives of many governments to promote equitable access to transportation infrastructure [123,124]. The improvements in bus travel times (25%) and emergency response times (30%) demonstrate the system’s capacity to deliver targeted benefits where they are most needed. However, achieving such outcomes requires addressing potential public resistance to rerouting strategies, particularly among private vehicle users [125,126,127]. Moreover, the benefits to cyclists and pedestrians, while implicitly included, could be expanded to ensure that all road users are equitably represented in traffic optimisation strategies.

Despite its limitations, this study contributes valuable insights into the practical applications of STM systems in urban settings. The findings reinforce the importance of predictive systems for addressing the dual challenges of congestion and sustainability, but they also highlight areas for improvement. Expanding the multiagent framework to incorporate additional variables, such as weather conditions or infrastructure disruptions, could further enhance its robustness and applicability. Pilot projects in diverse urban settings could also validate the scalability of the proposed framework and address any regional-specific challenges. Finally, studying the long-term behavioural shifts induced by these systems, such as changes in public transport usage or the adoption of active travel modes, could provide valuable insights for policymakers seeking to create more sustainable and equitable urban environments.

While the simulation framework utilised in this study is grounded in established traffic flow models and routing functions—including Poisson-based arrival processes and utility-driven decision rules—one limitation is the absence of direct validation using real-time IoT sensor data. Although the parameters were calibrated using publicly available traffic statistics, such as average travel speeds and congestion trends from Transport for London (TfL), no raw sensor feeds (e.g., from inductive loops or vehicle GPS traces) were integrated into the simulation. This limits the empirical generalizability of the results. Future work should seek to incorporate real-time IoT data sources to validate the simulation outputs against ground truth measurements and enhance the operational realism of the proposed framework.

## 6. Conclusions

The aim of this study was to explore the potential of IoT-enabled, sensor-driven adaptive traffic management systems for mitigating urban congestion, enhancing mobility, and reducing environmental impacts in densely populated cities. Focusing on London as a case study, the research utilised a multiagent simulation framework to model complex traffic scenarios and evaluate the effectiveness of innovative strategies, such as adaptive signal controls and dynamic rerouting.

Unlike conventional approaches that often rely on static traffic models or simplified assumptions, this study integrated real-time data from IoT sensor networks with a dynamic, multiagent-based simulation environment. This approach allowed for a nuanced representation of urban traffic systems, capturing the interactions among diverse road users, including private vehicles, buses, cyclists, and emergency services. The incorporation of predictive analytics further distinguishes this study from most other research in this domain by enabling proactive adjustments to traffic flows, such as pre-emptive signal timing and real-time rerouting, enhancing both efficiency and adaptability. These advancements position this study as a significant step forward in the field of urban traffic management.

The key findings demonstrate the efficacy of the proposed framework. The average travel times were reduced by 30%, the congestion levels at major intersections decreased by 50%, and CO_2_ emissions dropped by 28%. These results highlight the transformative potential of sensor-based adaptive traffic systems for achieving both operational efficiency and sustainability. Additionally, the system’s scalability and ability to address diverse traffic scenarios, including peak-hour congestion and special events, further reinforce its applicability to complex urban environments.

This study contributes to the current literature by addressing critical gaps in adaptive traffic management research. While many existing studies focus on limited geographic or temporal scopes, this research provides a comprehensive, scalable framework applicable to diverse urban contexts. Moreover, it emphasises equity and inclusivity by prioritising high-occupancy vehicles and emergency response units, aligning with broader policy goals to promote sustainable and equitable urban mobility.

The implications of this research extend to policymakers, urban planners, and transportation authorities. By adopting sensor-enabled adaptive systems, cities could optimise their traffic networks, reduce their environmental footprints, and enhance the overall quality of urban life. Furthermore, the framework developed in this study serves as a replicable model for other metropolitan areas, providing actionable insights to drive innovation in smart city planning.

In summary, this study not only demonstrates the technical feasibility of advanced traffic management systems, but also underscores their broader societal value. By bridging technological innovation with practical policy applications, it offers a robust pathway to addressing the pressing challenges of urban mobility and sustainability, contributing meaningfully to the evolving discourse on smart city development. However, this framework does not yet incorporate recent advances, such as deep reinforcement learning (DRL) or edge-computing-based optimisation, which offer enhanced adaptivity and decentralised control capabilities. Exploring these directions in future work could further improve the real-time responsiveness, energy efficiency, and operational scalability, strengthening the framework’s integration within next-generation smart city architectures.

## Data Availability

Data can be made available upon request to ensure privacy re-strictions are upheld.

## References

[1] Vellela S.S., Manne V.K., Trividha G., Chaithanya L., Shaik A. (2025). Intelligent Transportation Systems AI and IoT for Sustainable Urban Traffic Management. SSRN Electron. J..

[2] Čolaković A., Salihović N., Dželihodžić A. (2023). Adaptive Traffic Management Systems Based on the Internet of Things (IoT).

[3] Khang A., Singh K. (2025). Internet of Things (IoT) Smart Sensing Traffic Lights for Revolutionizing Urban Traffic Management.

[4] Salman M.Y., Hasar H. (2023). Review on Environmental Aspects in Smart City Concept: Water, Waste, Air Pollution and Transportation Smart Applications Using IoT Techniques. Sustain. Cities Soc..

[5] Sreelekha M., Midhunchakkaravarthy (2025). Revolutionizing Urban Traffic Management: IoT-Driven Algorithms for Intelligent Transportation Systems. Int. J. Intell. Transp. Syst. Res..

[6] Sahil, Sood S.K. (2021). Smart Vehicular Traffic Management: An Edge Cloud Centric IoT Based Framework. Internet Things.

[7] Pérez-Guzmán D., Fernández-Fernández M., Sánchez-Contreras J., Pérez R., Salgueiro Y. (2024). Control Network and Communications for Devices IoT Applied to Agroindustry. Proceedings of the 2024 IEEE International Conference on Automation/XXVI Congress of the Chilean Association of Automatic Control (ICA-ACCA).

[8] Shvedenko V., Shvedenko V.N. (2024). Ensuring a Harmonious State of Smart Space When There Is a Conflict of Interest of Its Elements. Smart Spaces.

[9] Hussain S., Ahonen V., Leviäkangas P. (2025). Sustainability Assessment of Smart Mobility Projects in Finland: A Comparative Analysis. Transp. Res. Procedia.

[10] Guix M., Nájera Sánchez J.J., Bonilla Priego M.J., Font X. (2025). The Changing Institutional Logics behind Sustainability Reports from the Largest Hotel Groups in the World in 2014, 2018 and 2021. Tour. Manag..

[11] Sathiyaraj R., Bharathi A. (2019). An efficient intelligent traffic light control and deviation system for traffic congestion avoidance using multi-agent system. Transport.

[12] Humayun M., Afsar S., Almufareh M.F., Jhanjhi N.Z., AlSuwailem M. (2022). Smart Traffic Management System for Metropolitan Cities of Kingdom Using Cutting Edge Technologies. J. Adv. Transp..

[13] Dui H., Zhang S., Liu M., Dong X., Bai G. (2024). IoT-Enabled Real-Time Traffic Monitoring and Control Management for Intelligent Transportation Systems. IEEE Internet Things J..

[14] Zhu F., Lv Y., Chen Y., Wang X., Xiong G., Wang F.-Y. (2020). Parallel Transportation Systems: Toward IoT-Enabled Smart Urban Traffic Control and Management. IEEE Trans. Intell. Transp. Syst..

[15] McKenney D., White T. (2013). Distributed and Adaptive Traffic Signal Control within a Realistic Traffic Simulation. Eng. Appl. Artif. Intell..

[16] Zhu X., Yan J., Qu Y. (2012). Distributed Consensus Algorithm for Networked Euler-Lagrange Systems with Self-Delays and Uncertainties. J. Syst. Eng. Electron..

[17] Mutambik I. (2024). An Entropy-Based Clustering Algorithm for Real-Time High-Dimensional IoT Data Streams. Sensors.

[18] Xu Y., Yin F., Xu W., Lin J., Cui S. (2019). Wireless Traffic Prediction With Scalable Gaussian Process: Framework, Algorithms, and Verification. IEEE J. Sel. Areas Commun..

[19] Li M., Wang Y., Wang Z., Zheng H. (2020). A Deep Learning Method Based on an Attention Mechanism for Wireless Network Traffic Prediction. Ad Hoc Networks.

[20] Abduljabbar R., Dia H., Liyanage S. (2025). Machine Learning Traffic Flow Prediction Models for Smart and Sustainable Traffic Management. Infrastructures.

[21] Kaššaj M., Peráček T. (2024). Synergies and Potential of Industry 4.0 and Automated Vehicles in Smart City Infrastructure. Applied Sciences.

[22] Ksibi S., Jaidi F., Bouhoula A. (2025). MLRA-Sec: An Adaptive and Intelligent Cyber-Security-Assessment Model for Internet of Medical Things (IoMT). Int. J. Inf. Secur..

[23] Zamri M.A., Hamzah N. (2022). The Implementation of Intelligent Traffic Management System in Solving Traffic Congestion: A Survey of Federal Route 3214. J. Phys. Conf. Ser..

[24] Nambajemariya F., Wang Y. (2021). Excavation of the Internet of Things in Urban Areas Based on an Intelligent Transportation Management System. Adv. Internet Things.

[25] Mutambik I. (2023). The Global Whitewashing of Smart Cities: Citizens’ Perspectives. Sustainability.

[26] Medina-Salgado B., Sánchez-DelaCruz E., Pozos-Parra P., Sierra J.E. (2022). Urban Traffic Flow Prediction Techniques: A Review. Sustain. Comput. Inform. Syst..

[27] Jiang W., Luo J. (2022). Graph Neural Network for Traffic Forecasting: A Survey. Expert. Syst. Appl..

[28] Zhou F., Yang Q., Zhong T., Chen D., Zhang N. (2021). Variational Graph Neural Networks for Road Traffic Prediction in Intelligent Transportation Systems. IEEE Trans. Industr Inform..

[29] Eze E.O., Keates S., Pedram K., Esfahani A., Odih U. (2022). An Event-Based Intelligent Vehicle Rerouting for Efficient Traffic Management for Connected Vehicles. Proceedings of the 2022 5th International Conference on Information and Communications Technology (ICOIACT).

[30] Garg T., Kaur G., Rana P.S. (2024). Traffic Rerouting Optimization Using Scheduling Algorithms. SN Comput. Sci..

[31] Peráček T., Kaššaj M. (2025). Legal Easements as Enablers of Sustainable Land Use and Infrastructure Development in Smart Cities. Land.

[32] Tiwari P. (2024). The Machine Learning Framework for Traffic Management in Smart Cities. Manag. Environ. Qual. Int. J..

[33] Chada S.K., Görges D., Ebert A., Teutsch R. (2023). Deep Learning-Based Vehicle Speed Prediction for Ecological Adaptive Cruise Control in Urban and Highway Scenarios. IFAC-PapersOnLine.

[34] Mutambik I., Lee J., Almuqrin A., Zhang J.Z. (2023). Transitioning to Smart Cities in Gulf Cooperation Council Countries: The Role of Leadership and Organisational Culture. Sustainability.

[35] D’Alfonso L., Giannini F., Franzè G., Fedele G., Pupo F., Fortino G. (2024). Autonomous Vehicle Platoons in Urban Road Networks: A Joint Distributed Reinforcement Learning and Model Predictive Control Approach. IEEE/CAA J. Autom. Sin..

[36] Elsagheer Mohamed S.A., AlShalfan K.A. (2021). Intelligent Traffic Management System Based on the Internet of Vehicles (IoV). J. Adv. Transp..

[37] Hira S., Hira S. (2024). Smart Energy Management Using Vehicle-to-Vehicle and Vehicle-to-Everything. Artificial Intelligence-Empowered Modern Electric Vehicles in Smart Grid Systems.

[38] Molinari F., Dethof A.M., Raisch J. (2019). Traffic Automation in Urban Road Networks Using Consensus-Based Auction Algorithms For Road Intersections. Proceedings of the 2019 18th European Control Conference (ECC).

[39] Nigam N., Singh D.P., Choudhary J. (2023). A Review of Different Components of the Intelligent Traffic Management System (ITMS). Symmetry.

[40] Li W., Chen J., Gao S., Niu L., Wei J., Sun R., Wei Y., Tang W., Cui T.J. (2024). An Externally Perceivable Smart Leaky-Wave Antenna Based on Spoof Surface Plasmon Polaritons. Opto-Electron. Adv..

[41] Walczak R., Koszewski K., Olszewski R., Ejsmont K., Kálmán A. (2023). Acceptance of IoT Edge-Computing-Based Sensors in Smart Cities for Universal Design Purposes. Energies.

[42] Sharif A., Li J., Khalil M., Kumar R., Sharif M.I., Sharif A. (2017). Internet of Things—Smart Traffic Management System for Smart Cities Using Big Data Analytics. Proceedings of the 2017 14th International Computer Conference on Wavelet Active Media Technology and Information Processing (ICCWAMTIP).

[43] Khalique A., Siddiqui F., Ahad M.A., Hussain I. (2024). Towards Trustworthy Urbanization: Security and Privacy Objectives for Sustainable Smart City.

[44] AlHalawani S., Benjdira B., Ammar A., Koubaa A., Ali A.M. (2024). DiffPlate: A Diffusion Model for Super-Resolution of License Plate Images. Electronics.

[45] Liu T., Mostafa S., Mohamed S., Nguyen T.S. (2020). Emerging Themes of Public-Private Partnership Application in Developing Smart City Projects: A Conceptual Framework. Built Environ. Proj. Asset Manag..

[46] Ionescu R.-V., Zlati M.L., Antohi V.-M. (2023). Smart Cities from Low Cost to Expensive Solutions under an Optimal Analysis. Financ. Innov..

[47] Dewitte P., Ausloos J. (2024). Chronicling GDPR Transparency Rights in Practice: The Good, the Bad and the Challenges Ahead. Int. Data Priv. Law.

[48] Ji A., He R., Chen W., Zhang L. (2024). Computational Methodologies for Critical Infrastructure Resilience Modeling: A Review. Adv. Eng. Inform..

[49] Orchi H., Diallo A.B., Elbiaze H., Sabir E., Sadik M. (2025). A Contemporary Survey on Multisource Information Fusion for Smart Sustainable Cities: Emerging Trends and Persistent Challenges. Inf. Fusion.

[50] Kuo Y.-H., Leung J.M.Y., Yan Y. (2023). Public Transport for Smart Cities: Recent Innovations and Future Challenges. Eur. J. Oper. Res..

[51] Mutambik I. (2024). Assessing Urban Vulnerability to Emergencies: A Spatiotemporal Approach Using K-Means Clustering. Land.

[52] Yıldırım Z.B., Özuysal M. (2024). Autonomous Vehicles and Urban Traffic Management for Sustainability: Impacts of Transition of Control and Dedicated Lanes. Sustainability.

[53] Šobot A., Gričar S., Bojnec Š. (2024). Sustainable Commuting: Active Transport Practices and Slovenian Data Analysis. Urban Sci..

[54] Mondal M.A., Rehena Z. (2021). An IoT-Based Congestion Control Framework for Intelligent Traffic Management System.

[55] Bellini P., Nesi P., Pantaleo G. (2022). IoT-Enabled Smart Cities: A Review of Concepts, Frameworks and Key Technologies. Appl. Sci..

[56] Dahal K., Almejalli K., Hossain M.A. (2013). Decision Support for Coordinated Road Traffic Control Actions. Decis. Support Syst..

[57] Seredynski M., Mazurczyk W., Khadraoui D. (2013). Multi-Segment Green Light Optimal Speed Advisory. Proceedings of the 2013 IEEE International Symposium on Parallel & Distributed Processing, Workshops and Phd Forum.

[58] World Bank (2021). Mobility and Development: Innovations, Policies, and Practices.

[59] Sayed S.A., Abdel-Hamid Y., Hefny H.A. (2023). Artificial Intelligence-Based Traffic Flow Prediction: A Comprehensive Review. J. Electr. Syst. Inf. Technol..

[60] Olawade D.B., Wada O.Z., Ige A.O., Egbewole B.I., Olojo A., Oladapo B.I. (2024). Artificial Intelligence in Environmental Monitoring: Advancements, Challenges, and Future Directions. Hyg. Environ. Health Adv..

[61] Mutambik I., Almuqrin A., Lee J., Zhang J.Z., Alomran A., Omar T., Floos A., Homadi A. (2021). Usability of the G7 Open Government Data Portals and Lessons Learned. Sustainability.

[62] Zhang Y., Li Y., Zhou X., Kong X., Luo J. (2019). TrafficGAN: Off-Deployment Traffic Estimation with Traffic Generative Adversarial Networks. Proceedings of the 2019 IEEE International Conference on Data Mining (ICDM).

[63] Manley E., Cheng T., Penn A., Emmonds A. (2014). A Framework for Simulating Large-Scale Complex Urban Traffic Dynamics through Hybrid Agent-Based Modelling. Comput. Environ. Urban Syst..

[64] Sayed S.A., Abdel-Hamid Y., Hefny H.A. (2025). Intelligent Traffic Flow Prediction Using Deep Learning Techniques: A Comparative Study. SN Comput. Sci..

[65] Bhouri N., Balbo F., Pinson S. (2011). Towards Urban Traffic Regulation Using a Multi-Agent System.

[66] Dai J., Li X. (2010). Multi-Agent Systems for Simulating Traffic Behaviors. Chin. Sci. Bull..

[67] Shih C.-Y., Chang C.-M., Wu B.-F., Chang C.-H., Hwang F.-N. (2024). Data-Driven Numerical Simulation with Extended Kalman Filtering and Long Short-Term Memory Networks for Highway Traffic Flow Prediction. J. Mech..

[68] Freiknecht J., Effelsberg W. (2017). A Survey on the Procedural Generation of Virtual Worlds. Multimodal Technol. Interact..

[69] Diallo A.O., Lozenguez G., Doniec A., Mandiau R. (2025). Utility-Based Agent Model for Intermodal Behaviors: A Case Study for Urban Toll in Lille. Appl. Intell..

[70] Shingate K., Jagdale K., Dias Y. (2020). Adaptive Traffic Control System Using Reinforcement Learning. Int. J. Eng. Res. Technol..

[71] Meyer M., Lejeune L., Giot C., Hay M., Bessot N. (2025). Sensitivity of Driving Simulation to Sleep Deprivation: Effect of Task Duration. Sleep.

[72] Chen D., Zhu M., Yang H., Wang X., Wang Y. (2024). Data-Driven Traffic Simulation: A Comprehensive Review. IEEE Trans. Intell. Veh..

[73] Chen L., Wu P., Chitta K., Jaeger B., Geiger A., Li H. (2024). End-to-End Autonomous Driving: Challenges and Frontiers. IEEE Trans. Pattern Anal. Mach. Intell..

[74] Pan L., Yang N., Zhang L., Zhang R., Xie B., Yan H. (2024). Assessment of the Impact of Multi-Agent Model-Based Traffic Optimization Interventions on Urban Travel Behavior. Electronics.

[75] Ju Z., Zhang H., Li X., Chen X., Han J., Yang M. (2022). A Survey on Attack Detection and Resilience for Connected and Automated Vehicles: From Vehicle Dynamics and Control Perspective. IEEE Trans. Intell. Veh..

[76] Zamanpour M., He S., Levin M.W., Sun Z. (2025). Incorporating Lane-Change Prediction into Energy-Efficient Speed Control of Connected Autonomous Vehicles at Intersections. Transp. Res. Part C Emerg. Technol..

[77] Arif M., Wang G., Zakirul Alam Bhuiyan M., Wang T., Chen J. (2019). A Survey on Security Attacks in VANETs: Communication, Applications and Challenges. Veh. Commun..

[78] Wang C., Quddus M.A., Ison S.G. (2013). The Effect of Traffic and Road Characteristics on Road Safety: A Review and Future Research Direction. Saf. Sci..

[79] Theofilatos A., Yannis G. (2014). A Review of the Effect of Traffic and Weather Characteristics on Road Safety. Accid. Anal. Prev..

[80] Saw K., Katti B.K., Joshi G.J., Kedia A. (2024). Travel Time Reliability Evaluation Using Fuzzy-Possibility Approach: A Case Study of an Indian City. Transp. Plan. Technol..

[81] Jamous W., Balijepalli C. (2018). Assessing Travel Time Reliability Implications Due to Roadworks on Private Vehicles and Public Transport Services in Urban Road Networks. J. Traffic Transp. Eng. (Engl. Ed.).

[82] Juhász M., Mátrai T., Koren C. (2017). Forecasting Travel Time Reliability in Urban Road Transpo. Arch. Transp..

[83] Chawla A., Khare M., Perugu H. (2024). Improving Exhaust Emission Evaluation: An Integrated Modelling Approach for Urban Road Networks in Diverse Operating Environments. Atmos. Pollut. Res..

[84] Afrin T., Yodo N. (2020). A Survey of Road Traffic Congestion Measures towards a Sustainable and Resilient Transportation System. Sustainability.

[85] Choudhary A., Gokhale S. (2019). Evaluation of Emission Reduction Benefits of Traffic Flow Management and Technology Upgrade in a Congested Urban Traffic Corridor. Clean. Technol. Environ. Policy.

[86] Ji J.S., Liu L., Zhang J., Kan H., Zhao B., Burkart K.G., Zeng Y. (2022). NO2 and PM2.5 Air Pollution Co-Exposure and Temperature Effect Modification on Pre-Mature Mortality in Advanced Age: A Longitudinal Cohort Study in China. Environ. Health.

[87] Mutambik I. (2024). The Sustainability of Smart Cities: Improving Evaluation by Combining MCDA and PROMETHEE. Land.

[88] Chun G., Rouphail N.M. (2025). A Movement-Based Delay Model for Signalised Alternative Intersections. Transp. A Transp. Sci..

[89] Hawkes A.G. (1966). Delay at Traffic Intersections. J. R. Stat. Soc. Ser. B Stat. Methodol..

[90] Al Suleiman S., Monzon A., Lopez E., Cortez A. (2025). Comparative Analysis of Perceived Quality of Bus Services in Different Socioeconomic and Cultural Urban Contexts: Tangier (Morocco) and Oviedo (Spain). Case Stud. Transp. Policy.

[91] Sogbe E., Susilawati S., Pin T.C. (2024). Scaling up Public Transport Usage: A Systematic Literature Review of Service Quality, Satisfaction and Attitude towards Bus Transport Systems in Developing Countries. Public Transp..

[92] Heumann M., Bäßmann F.N., Staritz J., Bienzeisler L., Wage O., Breitner M.H. (2024). Impacts of Sustainable Logistics in Urban and Rural Areas: A Delphi Study. Res. Sq..

[93] Damaj I., Al Khatib S.K., Naous T., Lawand W., Abdelrazzak Z.Z., Mouftah H.T. (2022). Intelligent Transportation Systems: A Survey on Modern Hardware Devices for the Era of Machine Learning. J. King Saud Univ.—Comput. Inf. Sci..

[94] Park J., Lee H., Park S. (2025). Development of Real-Road CO2 Emission Factors for Diesel Light-Duty Vehicles across Diverse Driving Conditions. Energy.

[95] Wang J., Dai H., Feng H., Guo M., Zylianov V., Feng Z., Cui J. (2025). Carbon Emission of Urban Vehicles Based on Carbon Emission Factor Correlation Analysis. Sci. Rep..

[96] Mutambik I., Almuqrin A., Alharbi F., Abusharhah M. (2023). How to Encourage Public Engagement in Smart City Development—Learning from Saudi Arabia. Land.

[97] Aldred R., Goodman A., Woodcock J. (2024). Impacts of Active Travel Interventions on Travel Behaviour and Health: Results from a Five-Year Longitudinal Travel Survey in Outer London. J. Transp. Health.

[98] Jeunen O., Goethals B. (2023). Pessimistic Decision-Making for Recommender Systems. ACM Trans. Recomm. Syst..

[99] Balog K., Zhai C. (2023). User Simulation for Evaluating Information Access Systems. Proceedings of the Annual International ACM SIGIR Conference on Research and Development in Information Retrieval in the Asia Pacific Region.

[100] Ekstrand M.D., Chaney A., Castells P., Burke R., Rohde D., Slokom M. (2021). SimuRec: Workshop on Synthetic Data and Simulation Methods for Recommender Systems Research. Fifteenth ACM Conference on Recommender Systems.

[101] Büchs M., Mattioli G. (2021). Trends in Air Travel Inequality in the UK: From the Few to the Many?. Travel. Behav. Soc..

[102] Office for National Statistics (2021). How the Population Changed in Westminster: Census.

[103] Cuenca Orozco N., Gutiérrez Madrid F., Quintero H.F. (2024). Metaheuristic Optimization of Agricultural Machinery for the Colombian Carnation Industry. Agronomy.

[104] Al-Heety O.S., Zakaria Z., Abu-Khadrah A., Ismail M., Shakir M.M., Alani S., Alsariera H. (2025). Traffic Congestion Control with Emergency Awareness and Optimized Communication Infrastructure Using Reinforcement Learning and Non-Dominated Sorting Genetic Algorithm. IEEE Access.

[105] Kalutharage C.S., Mohan S., Liu X., Chrysoulas C. (2025). Enhancing Automotive Intrusion Detection Systems with Capability Hardware Enhanced RISC Instructions-Based Memory Protection. Electronics.

[106] Singh A., Rathore H. (2025). Advancing Connected Vehicle Security through Real-Time Sensor Anomaly Detection and Recovery. Veh. Commun..

[107] Abdelkader G., Elgazzar K., Khamis A. (2021). Connected Vehicles: Technology Review, State of the Art, Challenges and Opportunities. Sensors.

[108] Maadi S., Stein S., Hong J., Murray-Smith R. (2022). Real-Time Adaptive Traffic Signal Control in a Connected and Automated Vehicle Environment: Optimisation of Signal Planning with Reinforcement Learning under Vehicle Speed Guidance. Sensors.

[109] Zhang J., Wang J., Zang H., Ma N., Skitmore M., Qu Z., Skulmoski G., Chen J. (2024). The Application of Machine Learning and Deep Learning in Intelligent Transportation: A Scientometric Analysis and Qualitative Review of Research Trends. Sustainability.

[110] Tang Y., Jin L., Ozbay K. (2023). How Does Driver Non-Compliance Destroy Traffic Routing Control?. Proceedings of the 2023 62nd IEEE Conference on Decision and Control (CDC).

[111] Shuki Y., Junpei T., Hiroyuki E., Naonori U. (2025). Searching Optimum Route with Variable Travel Time. IEEE Access.

[112] Wu F., Ye H., Bektaş T., Dong M. (2025). New and Tractable Formulations for the Eco-Driving and the Eco-Routing-and-Driving Problems. Eur. J. Oper. Res..

[113] Barth M., Boriboonsomsin K. (2008). Real-World Carbon Dioxide Impacts of Traffic Congestion. Transp. Res. Rec. J. Transp. Res. Board.

[114] El-Husseiny M., Mashaly I., Azouz N., Sakr N., Seddik K., Atallah S. (2024). Exploring Sustainable Urban Mobility in Africa-and-MENA Universities towards Intersectional Future Research. Transp. Res. Interdiscip. Perspect..

[115] Martinez L.M., Pritchard J.P., Crist P. (2024). Shared Mobility’s Role in Sustainable Mobility: Past, Present, and Future. Annu. Rev. Environ. Resour..

[116] Chaudhry A.-G., Masoumi H., Dienel H.-L. (2023). A Systematic Literature Review of Mobility Attitudes and Mode Choices: MENA and South Asian Cities. Front. Sustain. Cities.

[117] Stratigea A., Leka A., Panagiotopoulou M. (2019). In Search of Indicators for Assessing Smart and Sustainable Cities and Communities’ Performance. Smart Cities and Smart Spaces.

[118] Ismagiloiva E., Hughes L., Rana N., Dwivedi Y. (2019). Role of Smart Cities in Creating Sustainable Cities and Communities: A Systematic Literature Review.

[119] Xiaoyi Y., Boonyanmethaporn W. (2024). Research on the Design of Child-Friendly Landscape Space in Chengdu Crown Community Based on SDG. J. Lifestyle SDGs Rev..

[120] Taamneh M.M., Alomari A.H., Taamneh S.M. (2024). Using Machine Learning to Predict Pedestrian Compliance at Crosswalks in Jordan. Appl. Sci..

[121] Rafe A., Singleton P.A., Boyer S., Mekker M. (2025). Pedestrian Crossing Behaviors at Signalized Intersections in Utah: Factors Affecting Spatial and Temporal Violations. Int. J. Transp. Sci. Technol..

[122] Cai J., Wang M., Wu Y. (2024). Research on Pedestrian Crossing Decision Models and Predictions Based on Machine Learning. Sensors.

[123] Jin S.T., Kong H., Wu R., Sui D.Z. (2018). Ridesourcing, the Sharing Economy, and the Future of Cities. Cities.

[124] de Saboya R.T., D’Orsi E., Keivani R. (2024). Promoting Active Aging in Brazil: A Longitudinal Study of Land Use Mix and Utilitarian Walking in Older Adults. Cities.

[125] Barbon R.S., Madeira E.R.M., Akabane A.T. (2025). A Two-Context-Aware Approach for Navigation: A Case Study for Vehicular Route Recommendation. Ad Hoc Netw..

[126] Sharma S., Awasthi S.K. (2022). Introduction to Intelligent Transportation System: Overview, Classification Based on Physical Architecture, and Challenges. Int. J. Sens. Netw..

[127] de Souza A.M., Braun T., Botega L.C., Cabral R., Garcia I.C., Villas L.A. (2019). Better Safe than Sorry: A Vehicular Traffic Re-Routing Based on Traffic Conditions and Public Safety Issues. J. Internet Serv. Appl..

